# Deteriorated Vascular Homeostasis in Hypertension: Experimental Evidence from Aorta, Brain, and Pancreatic Vasculature

**DOI:** 10.3390/jpm12101602

**Published:** 2022-09-28

**Authors:** Hadi Taghizadeh, Ali Taghizadehghalehjoughi, Serkan Yildirim, Mustafa Ozkaraca, Sidika Genc, Yesim Yeni, Muhammed Yasser Mokresh, Ahmet Hacimuftuoglu, Aristidis Tsatsakis, Konstantinos Tsarouhas

**Affiliations:** 1 Tissue Mechanics Laboratory, Faculty of Biomedical Engineering, Sahand University of Technology, Tabriz 51335-1996, Iran; 2 Department of Pharmacology, Faculty of Medicine, Bilecik Seyh Edebali University, 11230 Bilecik, Turkey; 3 Department of Pathology, Faculty of Veterinary Medicine, Ataturk University, 25240 Erzurum, Turkey; 4 Department of Pathology, Faculty of Veterinary Medicine, Sivas Cumhuriyet University, 58140 Sivas, Turkey; 5 Department of Medical Pharmacology, Faculty of Medicine, Ataturk University, 25240 Erzurum, Turkey; 6 Department of Forensic Sciences and Toxicology, Faculty of Medicine, University of Crete, 71003 Heraklion, Greece; 7 Department of Cardiology, University General Hospital of Larissa, 41110 Larissa, Greece

**Keywords:** cardiovascular mechanobiology, smooth muscle cell, immature collagen, immunohistochemistry, biaxial tension test

## Abstract

Hypertension, as a primary risk factor for many fatal disorders, is prevalent in the elderly. There is wide literature on hypertension dealing with its biological and/or biochemical aspects; however, limited research is available on the multifactorial nature of hypertension from a mechanobiological standpoint. This study intended to study in parallel histopathological alterations and deviated protein expressions with the mechanical behavior of the hypertensive tissues. The Goldblatt (2K1C) method was chosen for induction of renovascular hypertension in rabbits. The microstructural and immunohistological characteristics of the aortic, pancreatic, and brain vasculature were investigated. The mechanical properties of the aortic tissue were also evaluated using biaxial tensile tests. Our findings indicated severe hypertrophy of the hypertensive vessels and declined content of intact smooth muscle cells. Most of the collagen I content of the wall was compromised and less functional type III collagen was highly expressed. Reversed collagen I to collagen III ratio was the main contributor to the hypertrophic and less stiff hypertensive vessel walls. The multifactorial nature of hypertension is illustrated, and smooth muscle cell detachment is identified as the sign of described degenerative cascades all along the arterial tree.

## 1. Introduction

Hypertension, manifested as consistent elevation of the blood pressure (BP) with serious implications in the cardiovascular system, is very common in ageing populations, is connected with the sedentary lifestyle of the modern world, and constitutes the primary risk factor for many secondary pathologies, leading to disability or even to death [1,2]. From atherosclerosis and aneurysm formation, leak or rupture in the cardiovascular system, to renal, gastrointestinal, corneal, and cognitive dysfunction (Parkinson’s and Alzheimer’s) [3,4], hypertension has been implicated in the pathophysiology both of the onset and the aggravation thereof, enhancing at the cellular level the transition of the quiescent epithelial cells to migratory and invasive mesenchymal cells [5]. Numerous mechanisms of BP regulation in the body uncover the multifactorial nature of hypertension. The onset of hypertension is primarily asymptomatic and remains undiagnosed until one of the secondary disorders is settled and clinical symptoms appear.

Cellular content of the arterial wall, i.e., endothelial and smooth muscle cells (SMCs), are very closely and accurately correlated with the levels of BP. When a BP value beyond the physiological range is “detected” by these cells, biochemical cascades of wall remodeling are activated [6]: signaling molecules and growth factors are released, reactive oxygen species prevail and protein expression is altered in the targeted cells, triggering synthesis and/or degradation of the extracellular matrix (ECM). These alterations are essential in order to reinforce the arterial tissue against excessive strain and probable dissection [7,8]. Similar examples of remodeling in connective tissues, e.g., bones [9], are well-documented. This remodeling pathway continues to depose ECM until the source cell is no longer experiencing loads and strains well above the physiological levels. When hypertension is characterized by established and permanent high BP, ECM deposition continues until hypertension-induced remodeling leads to hypertrophic enlargement of the vascular walls [10]. The physiological balance of the structure, and consequently the functionability of the SMCs, is compromised, resulting in higher apoptotic rates and altered adhesive properties of the cells to the ECM, altered cytoskeletal features, and vascular tone [11].

The cascades of mechanical stimuli transduction in arterial tissues are not fully understood; however, it has been shown that via the remodeling process, wall thickness is increased, and new ECM is deposited to restore the experienced stretches to the physiologic ranges [12]. It has been experimentally shown that a number of elastic lamellae remain unchanged in hypertension and the hypertrophic wall thickening is mostly due to the collagenous ECM deposition, although the quality of deposited ECM, especially from a biomechanical prospect, is not addressed [1,6]. The geometrical and mechanical rearrangement of the arterial wall, as a result of established hypertension, leads to the altered homeostasis between endothelial cells and SMCs described above, negatively affecting its response to the experienced strenuous mechanical environment and facilitating the onset of secondary pathologies, especially in the cardiovascular system [1].

Having briefly introduced these aspects of hypertension, the importance of bio-chemo-mechanical investigations of the affected vasculature, and an attempt to link such findings to the cellular and extracellular homeostasis, achieves both scientific and clinical added value. Respective studies include the implementation of animal models and the investigation of the microstructural, immunohistochemical and mechanical performance of healthy and hypertensive groups [13,14,15,16]. In the current study, a rabbit model of hypertension is used to investigate the mechanobiological homeostasis of the vascular tissue from the aorta, pancreas, and brain parenchyma using immunohistochemical assays and additional biaxial tensile tests of the aortic tissue. Healthy controls were used for comparison. Subsequently, the alterations were discussed from a biomechanical point of view.

## 2. Materials and Methods

### 2.1. Animal Model of Hypertension

Ten healthy New Zealand rabbits (male and 4–4.5 months-old), with an average weight of 3594 ± 472 g, were housed at 22 °C room temperature in the animal lab. The number of animals was kept minimal in accordance with previous studies [17,18,19] and following European Union regulations on animal welfare [20]. The animals had free access to food and water for one week before the start of the modeling procedure. The protocol of this animal study, including the hypertension model, surgical procedures, and immunobiological and mechanical testing of the biological tissues, was approved by the ethics committee of Ataturk University, Erzurum, Turkey (no. 36643897-000-E.1700160125).

The animals were randomly assigned to control and test groups. The Goldblatt (2K1C) method was chosen for the induction of renovascular hypertension in the latter group [21,22]. The animals were placed in deep anesthesia (ketamine 35 mg/kg+ xylazine 5 mg/kg) [23], the peritoneum was opened, and the right kidney was obstructed with an iron clamp in the lower part of the liver (renal artery). During the procedure, special attention was given to keeping the neighboring vessels undamaged during the surgical intervention. Next, the muscle and skin layers were stitched, and the animals were recovered. Metamizole (Novalgin^®^, 35 mg/kg) was used as a pain reliever, and anti-septic penicillin G (1500U) was injected intraperitoneally into the animals for one week. The same surgical, treatment and recovery procedure was performed on the control group used for comparison without attaching a clamp. The health status and heart rate of the animals were checked every three days. After this period, both groups were returned to normal housing conditions of 12 h light/dark cycles, a temperature of 22 °C, and unlimited food and water access. While the control group was supplied with normal water, normal saline was used for the hypertensive group. At the end of the first and second month, the BPs of the anesthetized animals (using ketamine 35 mg/kg + xylazine 5 mg/kg), both from the control and the test groups, were measured from the peritoneum (Vet-Dop2^TM^, Mill Creek, WA, USA).

### 2.2. Tissue Extraction and Preparation

At the end of the eighth week, all control and test animals were euthanized by administering an overdose of an anesthetic agent (inhalation of a mixture of 5% sevoflurane and oxygen); respiratory arrest occurred in 60 s, followed rapidly by decapitation. Next, rabbits were necropsied, and the intended tissue samples, i.e., pancreatic tissue, brain parenchyma, and aorta, were extracted. Pancreatic and brain tissues were directly taken into a 10% buffered formalin solution. Since the arterial samples were planned for additional mechanical tests, the descending part of the thoracic aorta, after the subclavian branching and up to the intercostal branching, was cleared, and the surrounding loose connective tissues were carefully removed. This particular part of the aorta (2–3 cm length) is exposed to mostly uniform hemodynamic loads, and therefore is characterized by consistent diameter (~6 mm) and microstructure [24], constituting an appropriate sample intended for mechanical and histological characterization. Narrow rings from each specimen were immersed in a fixative solution for histological evaluations, and the rest of the aortic tissues were submerged in phosphate-buffered saline (PBS) and stored at −18 °C.

### 2.3. Histopathological Examination

Fixed aortic, pancreatic, and brain vasculatures embedded in paraffin and 3 μm slices were cut by a microtome. These narrow tissue specimens were placed on glass slides for deparaffinization and staining. Hematoxylin–Eosin (H&E), Congo red, and Masson’s trichrome (MTC) stains were applied. Histopathological findings were evaluated as absent (−), mild (+), moderate (++), and severe (+++) [25]. The aorta wall thickness was measured in normal and test (hypertension) groups and expressed in µm. Two independent sets of thickness measurements were performed on fixed H&E slides and on unfixed specimens that were prepared for biaxial tests. The former was carried out by processing the microscopic images of the stained sections, while the latter was measured by a digital Vernier and the obtained value for each specimen was used in stress calculations, as described below.

### 2.4. Immunohistochemical Examination

Similar to the histological staining, narrow rings of the arterial tissues were cut, and after taking these samples on polarized (positively charged) slides, the deparaffinization process was applied. These slides were transferred to a 500-watt microwave oven for 15 min, and then treated with antigen retrieval (pH 6.0). They were subsequently washed with distilled water, and 3% H_2_O_2_ (for 10 min) to prevent endogenous peroxidase activity. After washing with PBS, samples were treated with Collagen I (Catalog no. Ab6308, Abcam, Waltham, MA, USA), Collagen III (Catalog no. NBP2-33328, Novusbio, Toronto, ON, Canada), and Alpha Smooth Muscle Actin (Catalog no. Catalog # GTX18147 with GeneTex, Los Angeles, CA, USA) primary antibodies at 37 °C for 15 min. The manufacturer-recommended procedure was followed using expose mouse and rabbit-specific HRP/DAB detection IHC (Catalog no. Ab80436, Abcam, Waltham, MA, USA) as the second kit. After the application of 3, 3′-Diaminobenzidine (DAP), which was used as a chromogen, the sections were passed through a series of graded xylol and were counterstained with Mayer’s hematoxylin.

### 2.5. Mechanical Tests

**Figure 1:**
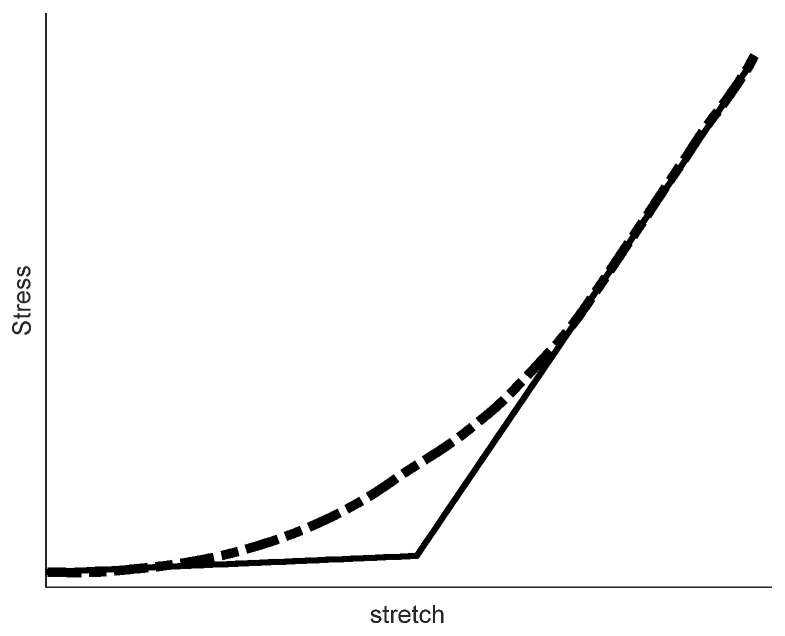
A typical stress-stretch curve of the arterial tissue samples is shown. The dashed line represents the: typical stress-stretch curve and the solid lines the initial and final curve tangents. The starting slope of the curve, E_init_, provides the stiffness of the ECM in crimped state of many collagen fibers). The tangent line of the linear region (in higher stretches), named E_fin_, demonstrates ECM stiffness with mechanical load-bearing of most of the collagen fibers. These two elasticity moduli were computed for the range of biaxial test data from the control and hypertensive groups.

On the test day, frozen aortic tissues were thawed at room temperature. The mechanical properties of the frozen soft tissue samples were shown not to be altered compared to the fresh specimens [26,27,28]. Next, the arterial cylinders were axially cut and opened to form a planar layout. Square samples (10 × 10 mm^2^) were prepared with their sides coinciding with the axial and circumferential directions of the artery (these directions denote the primary directions of the aortic tissue, as well). Sample thickness was measured on different sides via a digital Vernier, and the average of all measurements was incorporated in stress calculations exclusively. The mentioned square samples were tested biaxially in circumferential and axial directions. The obtained experimental force-displacement data were then converted to stress and stretch. Since the aortic tissue is exposed to large deformations and exhibits a nonlinear response, a respective continuum framework was adopted and the mechanical behavior of the tissue was reported in terms of Cauchy stress versus stretch ratio for each of the test directions. To quantify and compare the mechanical behavior of the axial and circumferential stress-stretch curves in control and hypertensive samples, values of the tangent modulus were obtained in the initial (λ ≤ 1.1) and linear (λ ≥ 1.4) regions of each stress-stretch curve. A sample stress-stretch curve and respective tangent lines denoting the initial (E_init_) and final (E_fin_) elastic moduli are shown in Figure 1. In low stretch ranges, collagen fibers are mostly crimped and the respective elastic modulus captures the mechanical behavior of other ECM components such as elastin and proteoglycans. The linear region of the curve is attributed to the engagement of nearly all of the collagen fibers; then, the respective tangent line denotes the stiffness of the overall collagen network with minor contributions from the non-collagenous ECM. Proposed elastic moduli provide quantitative measures of the fibrous ECM stiffness, and collagen content, for the control and hypertensive groups.

### 2.6. Statistical Analysis

The results were calculated as the mean ± standard error. The data were analyzed for normality and equality of variance as a precondition or justification for using parametric analysis. The low sample number did not fail the normality test. Statistical comparisons between groups were performed using one-way ANOVA with Tukey’s HSD test. For statistical analysis, all calculations were performed using IBM^®^ SPSS^®^ Statistics 20 software (IBM Corp., Armonk, NY, USA). Statistical significance was considered ^a^ *p* < 0.05 and ^b^ *p* < 0.001.

To determine the intensity of positive staining from the pictures obtained as a result of the dyeing, 5 random areas were selected from each image and evaluated in the ZEISS Zen Imaging Software program (ZEISS Group, Jena, Germany). Data were statistically defined as mean and standard deviation (mean ± Standard Deviation SD) for area %. The Mann-Whitney U test was performed to compare positive immunoreactive cells and immunopositive stained areas with the controls. As a result of the test, an *p*-value of <0.05 was considered significant and the data were presented as mean ± SD.

## 3. Results

### 3.1. Blood Pressure Measurement

Initial BP values, as well as readings at the 30th and 60th days, were recorded (Table 1). The background BP values range does not differ between the control and hypertensive group. BP values of the control group showed minimal variations during the experiment interval. On the contrary, in the hypertensive group, 11.3% and 15.2% increases are observed for the diastolic and systolic BPs, respectively, after 30 days. These changes become more profound on the 60th day, with an extra 5.60% and 13.1% increase of the diastolic and systolic BPs, respectively.

### 3.2. Histopathological Findings

**Figure 2:**
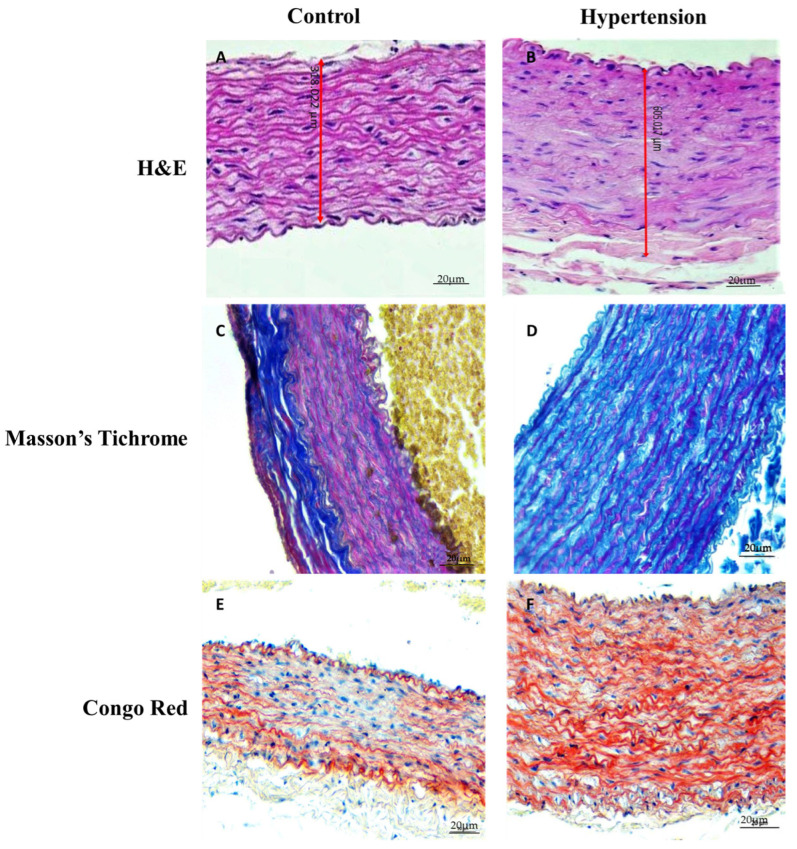
Histological stains of the aortic intima-media layers in samples collected from the control (first column) and the hypertensive group (second column). (**A**) H&E-stained section of the control group showing the thickness of the control sample and the circumferential tendency of the SMCs (in a “stretched” state) across the media. (**B**) H&E-stained section of the hypertensive group showing severe thickening of the media. Most SMCs have lost their circumferential tendency, appearing as round spots across the wall, in the hypertensive group. (**C**) Intact elastic sheets with adjacent collagen content and SMCs are evident on Masson’s trichrome stain of the control group. (**D**) Elastic sheet integrity is disrupted, and collagen fibers are substituted in these regions. Additionally, the SMC content of the wall markedly declined in the hypertensive group. (**E**) Congo red stain of the control group indicated no amyloid deposition. (**F**) Focal depositions of amyloid are noticed across the wall for the hypertensive group. Bar: 20 μm.

On the H&E-stained sections, severe thickening of the aortic wall of the hypertensive group was observed (318.022 µm for the control group and 605.017 µm for the hypertension group) (Figure 2A,B). Additionally, the circumferential tendency of the SMCs across the wall thickness is evident in the control group (white arrowheads, Figure 2A), while many of these cells do not have a stretched configuration in the hypertensive group, and seem likely to be detached from the neighboring ECM (Figure 2B, black arrowheads). As can be seen in Figure 2C, the lamellar units of the aortic media with the long and intact elastic sheets and adjacent collagen fibers are seen in the control group, while the elastic tissue in the hypertensive group is fragmented and replaced with collagenous tissue (black arrowheads, Figure 2D). Also, the SMC content observed in the control group (Figure 2C, pink color denotes SMC cytoplasm) is mainly declined in the hypertensive group as shown in Figure 2D. No evident amyloid deposition is seen in the normotensive group (Figure 2E), while there are some deposition sites across the hypertensive wall (black arrowheads, Figure 2F).

### 3.3. Immunohistochemical Findings

**Figure 3:**
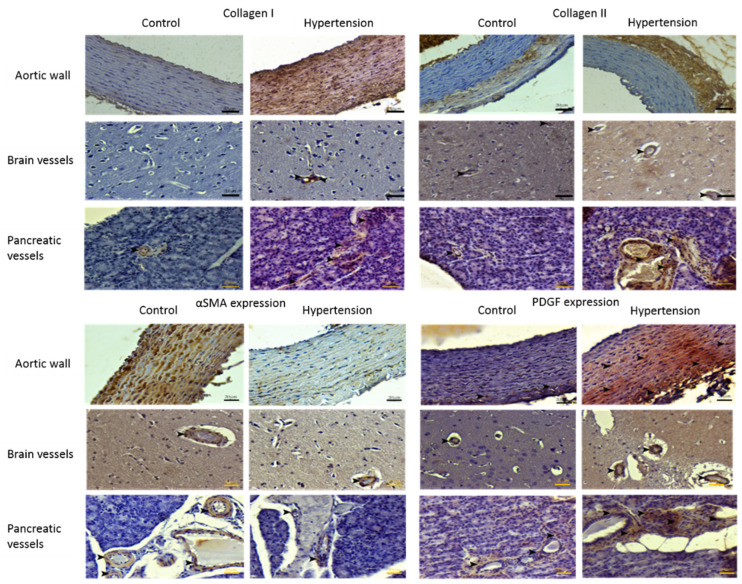
Immunobiological findings are hereby presented. Expression of collagen I in the examined tissues of the control and hypertensive groups (First and second columns, respectively). Severe expression of collagen I in the control aortic tissue and mild expression of this protein in the hypertensive group. Brain vasculature of the control group showed moderate expression of collagen I, compared to mild expression of collagen I in hypertensive parenchymal brain vasculature. For the normotensive controls, pancreatic vasculature expressed moderate collagen I, and mild expression of collagen I in pancreatic vessels of the hypertensive group is observed. Mild expression of collagen III in the aorta of the control group is observed. The hypertensive aortic tissue expressed type III collagen severely. Additionally, mild expression of collagen III was observed in the brain and pancreatic vasculature of the control group, and the hypertensive group demonstrated moderate collagen III expressions in the brain and pancreas. Differences in αSMA expression in the control and hypertensive groups were more noticeable in the aorta; the control group expressed αSMA severely, and the hypertensive group showed only mild expression. Brain and pancreas vasculature from controls showed moderate levels of αSMA expression, while the respective hypertensive group showed mild expression. For panels representing PGDF expression, arrowheads denote locations of focal expressions. Mild PDGF expression in outer regions of the aortic media for the control group against the severe expression of mentioned growth factor throughout the hypertensive aortic media was noticed. Additionally, for the brain and pancreatic vasculature, mild expression of PDGF in control and severe expression in the hypertensive group were noted. Black arrowheads are used to mention intended expression sites. IHC-P, Bar: 20 μm.

Expressions of collagen I and III as well as αSMA and PDGF (Platelet-derived growth factor) are depicted in Figure 3. Severe expression of collagen I was detected in the control group, while hypertensive aortae mildly expressed collagen I. In the brain vasculature, expression of collagen type I was moderate in the control group up to mild expressions in the hypertension group. In the case of pancreatic vessels, moderate expression of collagen I in the control group increased to mild levels in the hypertensive samples. Observations of the aortic tissue slides for the expression of collagen III revealed mild levels in the control group against severe expressions in the hypertensive group. The brain and pancreatic vasculature showed mild collagen III expressions in the control and severe collagen III expressions in the hypertensive groups. The pattern of αSMA expression in the aorta is markedly different compared to the brain and pancreatic vessels. Normotensive aorta samples expressed αSMA severely, while it was only mildly expressed in the hypertensive group. The expression levels in the brain and pancreatic vasculature in control were moderate. Mild αSMA expression in the brain and pancreatic vasculature of the hypertensive group was noticed. PDGF is slightly expressed in the control aortic tissue, and the respective hypertensive group demonstrated severe expression of this growth factor. Mild and severe expressions of PDGF are observed in the brain vasculature of the control and hypertensive groups, respectively. On the pancreatic tissue sections, mild expression of PDGF in the control group is observed, and the hypertensive group demonstrated severe PDGF expression. For ease of comparison, obtained data from immunohistochemical examinations are quantitatively represented in Table 2.

### 3.4. Biaxial Mechanical Tests

**Figure 4:**
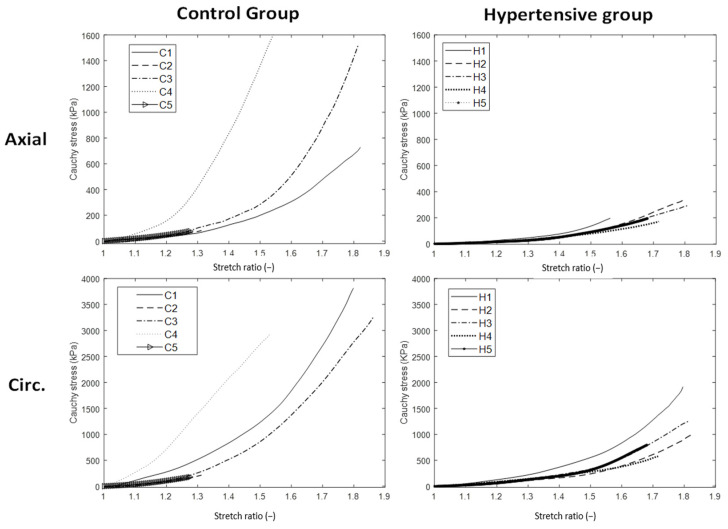
Stress-stretch curves for the control (first column) and hypertensive (second column) aortic samples in the axial (first row) and circumferential (second row) directions. For the range of tested specimens, a stiffer circumferential response is evident for both control and hypertensive groups. Comparing the direction-wise mechanical response between the control and hypertensive groups, higher stresses for the same stretch ratios were observed in the control samples compared to the hypertensive group. The levels of maximal circumferential stresses are approximately 2.5× of the maximal axial stresses. Next, for better demonstration and comparison, stress limits were differently set on axial and circumferential stress-stretch axes (1600 and 4000 kPa, respectively).

Figure 4 shows the stress-stretch curves for the mechanical response of the control and the hypertensive aortic tissues. A stiffer circumferential response compared to the axial stress-stretch curve is observed both in the control and in the hypertensive arterial samples. However, when the mechanical axial responses of the hypertensive aorta were compared to those of the control tissue, markedly stiffer axial and behaviors were noticed for the control group specimens; more specifically, at the stretch ratio of 1.8, axial stress for the control group was measured beyond 600 kPa, while in all specimens harvested from the hypertensive group, stress values were found to be more than 30% lower (below 400 kPa). Similarly, the circumferential response of the aortic tissues from the control group was found to be 30% higher than the respective samples from the hypertensive group (3000 kPa versus 1000 kPa, respectively). The calculated E_init_ and E_fin_ modules for the samples from the control and hypertensive groups are summarized in Table 3. Initially compliant mechanical response (minor stress elevation for increments of tissue stretch, i.e., small E_init_ values) is common to all test specimens and testing directions. When comparing the axial direction between the groups, the E_init_ for the control group is almost two times that of the hypertensive group. In the circumferential direction, E_init_ values are in the same range for the two groups. The J-shape and stiffening stress-stretch curve are also common to all test samples, but when we take a closer look at the respective E_fin_ values, axial moduli are markedly different (4962.2 ± 579.02 kPa for the control group versus 659.66 ± 142.37 in the hypertensive group). In addition, circumferential E_fin_ for the control group is 40% higher than the respective value of the hypertensive samples.

## 4. Discussion

In the present study, it has been shown that hypertension negatively affected both the cellular content and the extracellular matrix homeostasis of the analyzed vascular tissues. Immunohistological evidence showing fragmented elastic sheets and high collagen levels (Figure 2), and declined SMC content and SMC detachment from the neighboring ECM observed in the hypertensive group (Figure 3), support the conclusion that elevated levels of BP on the vascular tissue exert a degenerative effect both on its structure and its respective function. The presented data for the brain parenchyma and pancreatic vasculature (Figure 3, Table 2) indicates that the described degenerative process is not confined only to the main vasculature and the aorta, but it engages also peripheral arteries and the brain parenchyma, leading to deteriorated ECM composition, declined arterial mechanobiology, and anomalous function of the respective organs.

The distribution of SMCs across the tunica media, their contractile phenotype, and circumferential alignment turn into the primary regulators of arterial mechanobiology and homeostasis [29]. Circumferential alignment and stretched configuration of the SMCs in the control group (Figure 2A) indicate their healthy state and active role in the physiological behavior of the arterial wall. This orientation pattern is highly distributed in the hypertensive group (Figure 2B), which indicates that SMCs, as the primary ECM synthesizing cells, are decoupled from the ECM and the course of mechanical loading on the adjacent fibers. Markedly smaller αSMA expression in the hypertensive group indicates declined stress fibers that act as the load sensors for the SMCs [30], confirming the histological findings showing SMC detachment. The already mentioned degenerative cascade is in complete agreement with the observed fragmentation of elastic tissues in the hypertensive samples (Figure 2D), due to the exposure of arterial tissue to supraphysiological loads [8]. Collagenous tissue is deposited instead of fragmented elastic tissue (Figure 2D), and overall collagen content is increased in the hypertensive arterial wall compared to the control group. Nevertheless, due to the disturbed homeostasis of SMCs, the recently deposited collagenous content would remain immature, and protein synthesis of the arterial cells to restore the altered biomechanical loads back to normal levels becomes inefficient [31,32]. The detachment of SMCs from their respective ECM and increased transmural loads due to elevated BPs explain the excessive deposition of collagen fibers throughout the wall (Figure 2D). The reported accumulation of amyloid species in the hypertensive vasculature (Figure 2F) sheds light on the close association of hypertension with neurologic degenerations (especially Alzheimer’s disease), fibrosis and formation of atherosclerotic lesions in the brain and pancreas [33,34].

The remodeling process in the arterial media, which is primarily dependent on the SMC signaling pathways, tends to add more collagen to the wall to restore the mechanical environment of the SMCs to normal [35]. However, decreased collagen I expression in the hypertensive group aorta, along with severe expression of collagen III in this group (Table 2), is further evidence of immature and impractical collagen deposition in hypertension [32]. Such malformed fibrous ECM will progressively disturb cell-signaling pathways, and the resulting defective ECM mutually leads to increased deposition of defective fibrous content [33]. Comparing the reversed proportion of collagen I and III in hypertension (Table 2), one can infer that the level of collagen compromise in hypertension is directly correlated with the respective BP level in large and small arteries. Additionally, the observed decline in collagen I content and altered proportions of the collagen subtypes in hypertension agree with increased collagen I degrading enzymes in hypertension [36].

The αSMA positive control group (Table 2) demonstrates intact smooth muscle cells that contain actin stress fibers which are essential for the contractile and sensory functions of the SMCs across the arterial wall [37,38]. At the same time, the hypertensive vessels with less αSMA expression represent fewer intact SMCs to generate contractile force and contribute to arterial mechanotransduction. This phenomenon is correlated with the disappeared circumferential alignment of SMC content in the hypertensive group (Figure 2B). The mentioned decline of the SMC content and quality is closer to pathological phenotypes of degenerative arterial diseases, such as aneurysm and aortic dissection, rather than obstructive diseases such as atherosclerosis [38,39,40,41]. It is worth mentioning that hypertension is also a risk factor for plaque formation through focal amyloid depositions (Figure 2D). SMC degradation was modest for the brain and pancreatic vasculature (Table 2), implying that peripheral vessels are less damaged compared to the large conduit arteries near the heart.

PDGF expressions in the hypertensive group are independent of the artery size and location, since very high expressions for all of the tested vasculature are observed (Table 2). Previous findings indicate more than three times growth factor expression in the hypertensive samples [42]. It is shown that the mentioned upregulation might correlate with the increased levels of mechanical loading. Sensitive organs have specific protective mechanisms against hypertension. For example, brain circulation is shown to resist hypertension by regulative mechanisms such as microglial and fibroblast-dependent signaling pathways [43,44,45,46], which might explain minor degenerative changes despite the severe expression of growth factors in these tissues (Figure 3).

Elastin is the main mechanically relevant protein in the range of minor deformations of the arterial wall. Therefore, lower E_init_ values for the axial direction of the hypertension group (Table 3) indicate fragmented elastic tissue, which confirms our histological findings of lost elastic sheet integrity (Figure 2C,D). On the other hand, a similar range of E_init_ for the control and hypertensive groups in the circumferential direction indicates minor alterations in the elastic tissue network. Consequently, one can deduce that the degraded elastic tissue is significant in the axial direction and minimal in the circumferential direction. It can indicate directional degradation of elastic sheets, which should be more precisely addressed in future experimental investigations. The stiffening response of the aortic tissue (Figure 4) indicates the engagement of the collagen fibers. Mechanical properties are largely determined by the organization of collagen fibers. The stretch value corresponding to the onset of this nonlinear response is a measure of collagen waviness (less wavy collagen fibers, engage at lower stretches). It represents an efficient marker of arterial remodeling as mentioned in biomechanics literature [46,47]. The beginning of the nonlinear mechanical behavior for the tested samples occurs in stretch ratios of 1.3 and 1.4 for the control and hypertensive groups (Figure 4), respectively. Delayed involvement of collagen fibers in the hypertensive group indicates altered waviness/orientation of the deposited fibers as another marker of confused collagen synthesis in hypertension. The given values for the E_init_ and E_fin_ are in agreement with tangent modulus reports for the pre-atherosclerotic and aneurysmal tissue [48,49].

On the other hand, markedly stiffer axial and circumferential responses are observed in the control group compared to the hypertensive counterparts. Disturbed collagen balance in hypertension, as depicted by declined collagen I and increased collagen III content, is interconnected to the mechanical signals related to hypertension via the homeostatic activity of the SMC. Considering the existing evidence pointing to SMC inability in hypertension, constant ECM deposition signals are sent, but the deposited extra collagen content is immature (mainly type III collagen) (Figure 2D). Less stiff hypertensive tissue (especially the aortic tissue) confirmed reported histological and immunohistochemical findings.

### Limitations of the Study

The experimental design discussed in the present study provides an interesting insight into the mechanobiology of hypertension. Nevertheless, some limitations have been recognized. Namely, the animal population of the study was relatively small, serum renin activity was not measured and the hypertensive model was verified by measuring blood pressure, and only a dissection of a relatively long segment adjacent to the diaphragm without specifying the thoracic or abdominal part was harvested and analyzed after being defrozen. In addition, no quantitation of SMCs has been performed, apoptosis, detachment and deviation from the circumferential have not been monitored, and the gene expression levels of related genes were not evaluated.

## 5. Conclusions

Altered vascular microstructures and the respective signaling pathways were studied in an animal model of hypertension, using histological and immunobiological tests on samples of the brain parenchyma, pancreatic vasculature, and aortic tissues. This mechanobiological evaluation was complemented with biaxial tensile tests of the aortic tissues from control and hypertensive samples. While hyperplasia was observed in the fibrous matrix of the hypertensive samples, the atrophic appearance of the SMCs indicated their detachment from the ECM, which will eventually interrupt the homeostatic state of fiber deposition/degradation. These phenomena deteriorate the aortic mechanobiological equilibrium severely. On the other hand, the peripheral vasculature of the brain and pancreas are less impacted. Another interesting finding is that although the overall collagen content is increased in tissues studied from the hypertension model, deposited collagen I fibers are immature, and type III collagen is markedly increased. Therefore, the resulting mechanical behavior of the hypertensive vasculature decay significantly. Deposition of amyloid streaks, the altered quality and proportion of the collagen subtypes, and severe levels of growth factor expression decrease vascular flexibility and propose a secondary increase in blood pressure. Furthermore, it has been shown that the ECM stiffness, as depicted by E_init_ and the E_fin_, along with the stretch ratio of the nonlinear response onset, constitute a simple set of parameters that could address the mechanical behavior of healthy and diseased soft tissues. The findings of the present study provide a firm outline of the mechanobiology of hypertension underlining the multifactorial dimension thereof affecting the entire arterial tree. Therefore, monitoring quantitatively the ECM content in combination of the mechanical loads and tissue stiffness could lead to more comprehensive and focused treatment strategies beyond the existing schemes.

## Figures and Tables

**Table 1:** BP data of the control and hypertensive groups in different intervals during the animal modeling procedure.

Group	Background Values		30th Day		60th Day	
	Diastolic Pressure (mmHg)	Systolic Pressure (mmHg)	Diastolic Pressure (mmHg)	Systolic Pressure (mmHg)	Diastolic Pressure (mmHg)	Systolic Pressure (mmHg)
Control group	84.2 ± 4.71	115 ± 5.34	84.4 ± 7.05	115 ± 8.52	85.3 ± 5.54	118 ± 6.54
Hypertension Group	85.0 ± 3.23	115 ± 4.06	95.7 ± 9.04	**133 ± 7.02 ^a^**	98.0 ± 5.85	**152 ± 7.51 ^a^**

^a^ in bold statistical significance is denoted compared to control group *p* < 0.05.

**Table 2:** Scoring of immunohistochemical findings in the aortic, brain, and pancreatic vasculature. Since the responses of the brain and pancreatic vessels were similar, they were presented together. Significant differences were observed between the control and hypertensive group in all of the tested tissues (*p* < 0.05).

Parameter	Aorta		Brain and Pancreatic Vasculature	
	Control	Hypertension	Control	Hypertension
Collagen I	64.11 ± 4.22	20.24 ± 6.45 *	54.36 ± 5.71	19.23 ± 6.17 *
Collagen III	28.18 ± 3.54	49.18 ± 6.13 *	17.52 ± 5.18	35.0 ± 6.13 *
αSMA	62.71 ± 5.18	31.18 ± 4.12 *	40.13 ± 4.28	21.22 ± 5.18 *
PDGF	29.56 ± 4.18	63.74 ± 5.18 *	18.25 ± 6.75	60.11 ± 4.35 *

* in bold, statistical significance is denoted compared to control group *p* < 0.05.

**Table 3:** Calculated values of tangent moduli (E_init_, E_fin_) for the control and hypertension groups in axial and circumferential directions. The mean and standard deviation of the data groups are also provided.

Sample No.	Control				Hypertension			
	Axial		Circ.		Axial		Circ.	
	E_init_ (kPa)	E_fin_ (kPa)	E_init_ (kPa)	E_fin_ (kPa)	E_init_ (kPa)	E_fin_ (kPa)	E_init_ (kPa)	E_fin_ (kPa)
1	139.9	4983	493.1	7748	159.1	521.2	372.3	5704
2	201.5	4229	470.4	6696	97.62	882.9	261.3	5006
3	238.1	4991	379.2	7742	89.05	687.8	404.6	4821
4	172.5	5646	387.1	6734	52.05	553.7	429.4	5093
5	193.2	N/A	329.9	N/A	84.94	652.7	353.5	4936
mean ± sd	189.0 ± 36.29	4962 ± 579.0	411.8 ± 67.81	7230 ± 594.9	96.54 ± 39.02	659.7 ± 142.4	364.2 ± 64.52	5112 ± 345.6

## Data Availability

The datasets used and/or analyzed during the current study are available from Bilecik Seyh Edebali University, Faculty of Medicine, Department of Pharmacology upon reasonable and justifiable request, following the rules and procedures of the said University.
